# Integrating mental health into primary care for post-conflict populations: a pilot study

**DOI:** 10.1186/s13033-016-0046-x

**Published:** 2016-02-27

**Authors:** Chesmal Siriwardhana, Anushka Adikari, Kaushalya Jayaweera, Buddhika Abeyrathna, Athula Sumathipala

**Affiliations:** Global Public Health, Migration and Ethics Research Group, Faculty of Medical Science, Anglia Ruskin University, Chelmsford, CM1 1SQ UK; Institute of Psychiatry, Psychology and Neuroscience, King’s College, London, UK; Institute for Research and Development, Sri Jayawardenepura Kotte, Sri Lanka; Department of Psychiatry, Faculty of Medicine and Allied Sciences, Rajarata University of Sri Lanka, Mihintale, Sri Lanka; Research Institute for Primary Care and Health Sciences, Keele University, Keele, UK

**Keywords:** Sri Lanka, Post-conflict, Mental health, Primary care, mhGAP

## Abstract

**Background:**

Mental health care in post-conflict settings is often not prioritized, despite its important public health role. There is a salient gap in integrating mental health into primary care, especially in post-conflict settings. In the post-conflict Northern province of Sri Lanka, a pilot study was conducted to explore the feasibility of integrating mental health into primary care through a mhGAP-based training intervention.

**Methods:**

Using the mhGAP training intervention modules, a 24 h training programme was held over 3 days for primary care practitioners serving post-conflict populations (including internally displaced people and returnees). mhGAP intervention guide and video material was used in the training. Pre/post knowledge increase was measured. A qualitative study was also nested within the training programme to explore views, attitudes and perceptions of primary care practitioners on integrating mental health into primary care in the region. In-depth interviews were conducted.

**Results:**

Twelve primary care practitioners participated. The average service duration of the group was 7.6 years. The mean pre- and post-test scores of the PCP group were 72.8 and 77.2 % respectively. All 12 took part in the qualitative component. Participants highlighted their experiences of conflict and displacement, discussed the health profiles/needs of post-conflict populations in the region and provided insight into mental health care and training needs at primary care level. Participants also provided feedback on the mhGAP-based training; the cultural and contextual relevance of training material and content.

**Conclusion:**

This study was planned as a local demonstrative project to explore the feasibility of training primary care practitioners to promote the integration of mental health into primary care for post-conflict populations. To our knowledge, this is the first such attempt in Sri Lanka. Findings highlight the practical, operational and attitudinal barriers to integrate mental health into primary care, especially in resource-poor, post-conflict settings. Important feedback on mhGAP intervention guide, its implementation and training material was gained.

## Background

Mental health care is often given a secondary priority in post-conflict settings, where resources are usually allocated for more immediate health concerns despite the clear evidence available about increased mental disorder burden amongst post-conflict populations [1]. For most post-conflict populations living in resource-poor settings, even basic mental health treatment can only be accessed through primary care services. However, there is a salient gap in integrating mental health into primary care systems in post-conflict settings, due to lack of human resources, training, financial constrains, political will, stakeholder engagement and the lack of knowledge/awareness of policy makers [2, 3].

Sri Lanka, an island nation in South Asia, had experienced three decades of civil conflict, mainly centered around its Northern and Eastern Provinces. These regions, consisting of internally displaced people (IDP) and returnee forced migrants, have reported increased rates of mental disorders [4–8]. The post-conflict areas of Sri Lanka, despite an effective primary care system in place, has a significant treatment gap for mental health [9, 10]. The existing approach to providing mental health care to the conflict-affected population in Sri Lanka is through limited numbers of Medical Officers of Mental Health (numbering 1 MOMH to 30 villages or more) and mental health units at regional hospitals and a psychiatric unit at the Jaffna Teaching Hospital [11]. These resources are insufficient to meet the population needs, especially due to the severity of trauma, difficulties experienced in displacement and return migration. Primary care practitioners (PCP) in the region, both at government of private medical facilities, regularly spearhead the mental health care efforts. While PCPs are perhaps the ideal health workforce members to deliver mental health care in such low-resource regions, they do so without adequate training, skills or support required to identify and treat mental disorders in busy primary care settings. Since the end of conflict in 2009, Northern Sri Lanka has seen a rapid rehabilitation and rebuilding process in health and other infrastructure [10]. However, the need for human resources is the least being met, as the rate of capacity building and training new cadres are significantly below that of the growing need in the region.

In this paper, we report findings from a pilot, feasibility study conducted in the Northern Province of Sri Lanka, with the main aim of integrating mental health into primary care through a training intervention for PCP providing services to post-conflict populations [3]. The training intervention, based on World Health Organization’s mental health Gap Action Programme and Intervention Guide (WHO mhGAP IG) [12], was a local demonstration project seeking to gather data on PCP engagement from post-conflict regions, suitability and implementation of mhGAP training and evaluation of pre/post training knowledge increase. Our study also aimed to add evidence to the global attempts of mhGAP IG implementation and our approach of using PCP to deliver mental health treatment at primary care level is in line with the task-shifting approach in the current global mental health field [13].

## Methods

The full study protocol has been published previously [3]. Therefore, only a summary of key methodological information is provided in the current manuscript to avoid replication. The primary objective was to test the feasibility of improving/increasing of identification, treatment and referral of CMD by PCP among post-conflict populations in resource-poor, rural primary care settings of northern Sri Lanka using a manualised, mhGAP-based training programme [12]. The secondary objective of the study was to explore the attitudes and perceptions of PCP on integrating mental health into primary care [3].

### Pilot intervention based on mhGAP

The study setting was Northern Province of Sri Lanka, affected by the 30 year civil conflict with displaced and returning IDPs. Initial recruitment of PCP for the pilot study commenced in early 2014. As per the study protocol, monitoring of their current practices regarding mental health patients commenced immediately upon recruitment. Ten PCP were recruited within a period of 3 months. However, due to several hitherto unperceived operational challenges such as lack of support from administrative bodies, lack of interest from PCP, difficulties in participant retention, geo-political issues in northern Sri Lanka, lack of locally relevant training resources and financial constrains (discussed later in detail) that arose during the initial recruitment period, recruitment progress was curtailed. Mitigation of these challenges was a complex task, without compromising the rigour of the study. A strategy re-think was conducted and a new study plan was formed by adapting relevant components to match operational challenges, without deviating from the original protocol [3]. It was decided to deliver the training to a maximum possible number of PCP in the region, and measure knowledge/skills change through pre/post evaluation. The purpose of delivering the training was to attempt to generate some level of interest among the PCP and administrative bodies about the initiative, and to emphasize the importance of mental health in primary care settings. The differences between original protocol [3] and new study plan are detailed in Table 1, to enable a clear comparison.

**Table 1:** Differences between the original protocol and pilot study

Component	Protocol	Pilot study
Design	Pilot randomized controlled trial (RCT) and a qualitative study	No RCT, qualitative study conducted
Participants	n = 86 PCPs (43 intervention, 43 control); patients also included for RCT	n = 12 PCPs for the training and the qualitative study
Inclusion criteria for participants	Fully registered with the Sri Lanka Medical Council, providing service mainly to IDPs through government or private clinics	Fully registered with the Sri Lanka Medical Council, providing service mainly to IDPs through government or private clinics
Measurements	PHQ, CSRI from patients	None as no patients were recruited
Duration of training	5 days, 8 h/day	3 days, 8 h/day
Training personnel	mhGAP-trained trainer (PI), local psychiatrists	mhGAP-trained trainer, local psychiatrist
Training modules	Depression, medically unexplained symptoms, suicide and alcohol/drug abuse	Depression, stress-related disorders, medically unexplained symptoms, suicide and alcohol/drug abuse
Monitoring and Evaluation	3 months prior to training, 3 months after training, 6 months after training	No monitoring pre or post-training

The training, based on the WHO mhGAP IG, was delivered in December 2014 as the key component of the revised study plan. The training was delivered by a mhGAP-trained trainer and a local psychiatrist with clinical and research experience. Key mhGAP IG modules (depression, stress related disorders, medically unexplained symptoms, alcohol/drug disorder and suicide), considered relevant to the study setting based on previous research evidence indicating priority mental disorders, contextual factors (conflict-related) [3–10] and participant background were selected and delivered in the training. The mhGAP IG and other relevant course material including videos developed by the WHO were used. The original mhGAP training schedule was adapted to fit the delivery of 6 modules within a 3 full-day training programme totaling 24 h of face-to-face delivery. WHO mhGAP pre- and post-training evaluations were used to measure knowledge increase among the participants. Feedback was gathered after each module delivery. Language of delivery was English, and training material was also in English language. Videos had English subtitles.

### Qualitative study on integrating mental health into primary care

A small-sample qualitative study nested within the pilot RCT was planned to explore the attitudes and perspectives of PCP on the importance of integrating mental health into primary care. However, given the strategic changes explained above, the qualitative component was incorporated into the training programme by conducting the interviews with those who took part (no undue influence was exerted, potential participants were notified that although the training was part of a research project, they had the right to refuse the research component and only receive training). This approach was the only way of ensuring that the project was carried forward. An in-depth individual interview based on a topic guide was conducted by three trained qualitative researchers to elicit views, perceptions, and attitudes of PCP towards mental health, importance of providing mental health care, integration of mental health into primary care settings, perceived barriers and requirements for such initiatives, and other related topics. Interviews were conducted in English and Sinhala, ensuring participant privacy, and were audio-recorded with consent. Audio recordings were transcribed verbatim, and analyzed using thematic and content analytic approaches.

Ethics approval was obtained from the Ethics Review Committee, Faculty of Medicine, University of Sri Jayawardenepura (#730/13). The study protocol was registered with the Sri Lanka Clinical Trial Registry (SLCTR/2013/025). Collaboration was established with the Ministry of Health, Northern Provincial health authorities and Government Medical Officer’s Association of Sri Lanka. Informed written consent was obtained from individual PCP for the training intervention. Separate informed written consent was obtained for the qualitative component.

## Results

### Training intervention

Twelve PCP from the Northern Province of Sri Lanka participated in the training during the 3 days. Table 2 describes the participant characteristics including gender distribution, education and service-related information. All PCP were physicians. Mean age of the 12 participating PCP was 43.7 (SD; 8.5). Only 2 female PCP participated, and only 2 had some kind of post-graduate medical education (non-mental health related). Average total service duration and service among IDP was 7.6 and 5.1 years respectively. On average, each PCP treated 62 patients from IDP communities per day in their clinics, based at divisional hospitals, central dispensaries and primary care units.

**Table 2:** Characteristics of PCP receiving the training intervention

Participant characteristic	N = 12
Gender	
Male	10
Female	2
Education	
Basic degree only	10
Post-graduate education	2
Main service affiliation	
Government	12
Private	0
Type of primary care facility	
Hospital (district/provincial)	3
Central dispensary	5
Other	4
Service duration	
Less than 5 years	5
5–10 years	4
More than 5 years	2
Service with IDP	
Less than 5 years	6
More than 5 years	5
Number of IDP patients seen per day	
Less than 50	4
Between 50–100	4
More than 100	2

According to participant PCP, most common physical illnesses encountered in primary care included fever, back/joint pain, generalized body/musculoskeletal pain, hypertension, diabetes, respiratory disease including asthma and respiratory tract infections, urinary tract infections, physical injury including road traffic accidents, diarrhoea and other gastrointestinal disease. Most common mental and neurological illnesses encountered in primary care included anxiety, depression, somatic pain and other symptoms, PTSD, psychoses, sleep disorders, alcohol use disorders, epilepsy and dementia.

A series of questions were asked from PCP about recognizing, diagnosing, managing and referring people with mental illnesses in their primary care practice, about post-referral services, importance of referrals, adequacy of their current training and ability to provide mental health care, educational and skills development opportunities, level of support from communities they serve and if PCP are satisfied with the adequacy of mental health care they currently provide. Table 3 provides a summary of their responses. Finally, each module was evaluated through a questionnaire and the overall knowledge increase was measured using the mhGAP pre/post tests. The mean pre- and post-test scores of the PCP group were 72.8 % (SD; 11.4) and 77.2 % (SD; 11.8) respectively, and the pre/post score difference was statistically not significant (paired t test; t(9) = −1.408, p = 0.193).

**Table 3:** Mental health at primary care level—current practices of study participants

Question	Response	Frequency (%)
How difficult do you find it to recognize mental illnesses?	Very difficult	0 (0)
	Difficult	1 (8.3)
	Neutral	6 (50)
	Easy	4 (33.3)
	Very easy	0 (0)
	No idea	1 (8.3)
How difficult do you find it to diagnose a mental illness?	Very difficult	1 (8.3)
	Difficult	3 (25)
	Neutral	4 (33.3)
	Easy	3 (25)
	Very easy	0 (0)
	No idea	0 (0)
How difficult do you find treating and managing a person with a mental illness?	Very difficult	1 (8.3)
	Difficult	7 (58.3)
	Neutral	3 (25)
	Easy	0 (0)
	Very easy	0 (0)
	No idea	1 (8.3)
How often do you refer a patient with mental illness for specialist psychiatric care?	Never	0 (0)
	Rarely	1 (8.3)
	Sometimes	6 (50)
	Often	3 (25)
	Always	2 (16.7)
If and when you refer a patient with a mental illness do you get feedback from either the specialist or the patient?	Yes	7 (58.3)
	No	5 (41.7)
Do patients who already diagnosed and getting treatment from a mental health specialist visit you for continuation of that treatment or any other health issues?	Yes	8 (66.7)
	No	4 (33.3)
How important do you think is to refer a patient with mental illness that you find difficult to manage for specialist psychiatric care?	Not at all important	1 (8.3)
	Low importance	0 (0)
	Slightly important	0 (0)
	Neutral	1 (8.3)
	Moderately important	0 (0)
	Very important	6 (50)
	Extremely important	4 (33.3)
Do you think you have sufficient training and ability to provide adequate mental health care to patients with mental illnesses?	Strongly disagree	1 (8.3)
	Disagree	2 (16.7)
	Somewhat disagree	3 (25)
	Neutral	2 (16.7)
	Somewhat agree	3 (25)
	Agree	0 (0)
	Strongly agree	0 (0)
How often do you get opportunities to upgrade your skills and knowledge in providing mental health care?	Never	2 (16.7)
	Rarely	6 (50)
	Sometimes	2 (16.7)
	Often	1 (8.3)
	Always	0 (0)
What level of support do you receive from the IDP community for your practice?	Poor	4 (33.3)
	Fair	2 (16.7)
	Good	3 (25)
	Very good	2 (16.7)
	Excellent	0 (0)
Do you think that you provide adequate mental health care to match the needs of the IDP community that you serve?	Never	0 (0)
	Rarely	4 (33.3)
	Occasionally	1 (8.3)
	A moderate amount	6 (50)
	A great deal	0 (0)

### Qualitative component

All 12 PCP taking part in the training consented to the qualitative component. Key themes emerging from data analysis highlighted the importance of providing mental health care for post-conflict populations, importance of mental health training interventions, perceived barriers and requirements for the integration of mental health into primary care (quotations provided have been edited for language clarity and any information even remotely linked to a possible identification of an individual has been removed).

#### Experiencing conflict and displacement

Some PCP stated that they themselves had been displaced during the conflict, and had to deal with their personal trauma while providing healthcare during the conflict. One participant had to leave the region as a refugee due to threats from armed groups, who had returned a decade later to finish medical education.

*“I entered the university in 1983….started education and then I had some problems with some groups and left the place, again I started my studies around 1996 I think, I finished my education, I started my career as a medical officer. Later I went to [place name] to work in the resettlement programme as a medical officer”*

Others had been providing healthcare services in the region for several years, including the period leading to the end of conflict, which saw an increased level of violence and forced displacement. Subsequent to the cessation of conflict, they had worked with displaced populations living in IDP camps and continue to work with returnee IDPs in their current roles.

*“During [that] period….myself and another doctor, two of us were working in the critical time of war in [place name]. Now I conduct regular clinics in post*-*conflict areas”*

*“When I moved to [place name] I could see lot of IDP patients. That time I went there to work, the people were not yet resettled so I worked with IDPs, who were displaced from conflict areas”*

*“My first appointment was to a hospital in [place name], held by armed groups, no one liked to go and work there, I worked there for more than 2* *years, then due to increased level of war, the road was closed, and hospital had to be closed, I was also responsible for two other small clinics at the same time”*

### Health profiles and needs of post-conflict populations

The PCP provide basic healthcare services to over a million people living in the Northern Province. Majority of this population has been exposed to decades of conflict-related violence and displacement. Some had been living in displacement for a long period of time, in areas bordering the conflict zone or other areas of the country and have only recently returned. Socio-demographic profiles of the population served by PCP mostly include farmers, fishermen and low-skilled manual labourers in rural areas. According to PCP, urban populations consist more of people with higher education levels and in skilled jobs.

*“They are a low socio economic level community… less education…multiple partners and marriages at young ages are issues”*

*“Every family has more than five or six children…they have big families but they are living in small houses…. education level is low, especially among females….some have children at a very young age”*

The health needs of the population has wide variations, according to the PCP. However, at the same time, they identified common health issues across the population, regardless of the level of war exposure, displacement status, age or gender. These include both physical and mental health issues. Physical conditions include cardiovascular conditions, respiratory disease, generalized body pain and physical injuries. Mental health conditions are dominated by somatic complaints, depression, anxiety and symptoms of psychological distress. Alcohol and substance use disorders are, according to PCP, on the rise in communities they serve.

*“Among physical disorders, diabetes is common along with hypertension. Skin diseases are commonly seen and infectious disease like diarrhea is common probably because people use common water sources (water wells) in the area there”*

*"Once I met a man (who has retired from his service) who came to me as he couldn’t eat and sleep well. I noticed this is because he was depressed"*

*“most patients present with somatization of their mental problems……medically unexplainable…..chest pain and back aches, knee joint pains. But when we examine them physically they actually don’t have any physical issue. But they are somatizing their stress and depression and it should be addressed by psychosocial support than prescribing analgesics….which can bring long term problems to them”*

The PCP noted that females may be more affected by mental ill health in the communities they serve, and that mental health related stigma strongly prevents people from seeking treatment and support at the primary care level. They also noted that mental health problems may not be diagnosed correctly at primary care level, and even when diagnosed, they do not have the means to offer services such as counselling or other forms of psychotherapy.

*“I think they are not getting proper treatment to their mental health problems, especially females…females have lot of family work and caring for children…..husbands may not support much”*

*“The communities don’t accept mental illnesses… to them, mental illnesses are related to religious or some other beliefs”*

*“..the stigma…is a multi*-*factorial thing. There should be a lot of changes…..in culture and the way of thinking of people that we can’t do within ten or twenty years. But we can give adequate privacy and….adequate trust….between clinicians and patients. We can overcome this stigma affecting the patients and get them treatment”*

*“because at our primary health care unit we don’t diagnose mental health problems always. Even if we suspect any mental health problems or any medically unexplained problems, people feel stigmatized and are reluctant to go to a psychiatrist. Even for counseling people want to avoid going to a psychiatrist (psychotherapist) because of the stigma”*

*“We think they are normal from outside appearances and their daily living. But, the loss of family members, loss of work lead to deeper problems in people”*

#### Mental health care and training needs in the region

The participants indicated a lack of resources and sometimes motivation to address mental health issues among IDP populations they serve.

*“Most of the people are suffering from some type of mental health problem. But there is no way to explore these people or a way to offer help such as counselling to them at this level”*

*“We should have some people trained in mental health at primary care level and if that facility is available, it is better than referring straight to psychiatrists”*

*“We need resources to solve these problems. Because it is difficult to find resources, and difficult to face these problems, most of us (PCP) are avoiding that (treating mental health patients) at our level. If we have any proper plans only then we can continue programmes to help these people in a sustainable way”*

*“It is a necessity to look into mental health needs of the people at first contact level…..but nowadays mental health treatment is available at tertiary levels after referrals. But people don’t screen or search for mental health problems at primary care level. So in that way we are missing a lot of mental health problems and prescribe unnecessarily analgesics and other drugs”*

Increased patient loads at primary care level was seen as a major barrier in effective identification and treatment of mental disorders.

*“Due to the patient load most of the PCP are spending around ten to fifteen minutes for each patient. During that period….they can’t build a doctor*-*patient relationship, good rapport. To find out or diagnose mental health problems we need more time than that. Practically it is difficult to spend that much of time with each patient, managing time is very difficult”*

*“Because our Out Patient Department (OPD) flow is around 85*-*90 patient per day, time is limited. And well trained medical officers are not available enough. The consultant psychiatrist is based at the teaching hospital [place name] which is very far away from this place (around 40* *km)”*

The need for locally relevant training material was an issue that emerged during the training. The WHO training material, while containing general and essential information, was felt lacking in conflict-related, culturally-specific experiences and contextual applicability. PCP felt that the effectiveness of the training would be enhanced by using local-language, culturally and contextually relevant video material. The PCP indicated the need for shorter, multiple training sessions (refresher training) and continuous monitoring/support. Group activity and role plays were positively received.

*“Better to give this type of mental health training in the mother tongue. If so, empathic communication skills will improve”*

*“Duration of modules (or lessons) is not sufficient. In future we have to increase the lesson time and increase participant numbers. I think that the group activity, role play medical history taking and group discussions are good and helps to share the experiences”*

*“Better to give frequent workshops regarding the mental health and how to diagnose or how to assess the patients to primary care practitioners….how to communicate, consultation models, how improve doctor patient relationship should be taught”*

*“This type of training can be considered as an introduction. But this is not sufficient enough. This can be used as an eye opener…. but……further structured, more intensive and regular training should be made available at primary health care level”*

Participants highlighted the lack of mental health training interventions in the post-conflict Northern Province. The PCP said that training programmes such as the COMGAP programme, would have benefitted them few years ago, when they were dealing with the acute aftermath of conflict and displacement. The PCP were of the opinion that other groups such as public health professionals, public health midwives, hospital workers including nurses/other staff and social care workers should also be provided similar training, to enhance community outreach, to increase mental health awareness, to reduce stigma and to increase the detection of mental health issues at the community level. Development of uniform guidelines and policies on delivering mental health treatment at primary care level was recommended.

*“First we need to improve the awareness about mental illnesses in the community….after that community may realize the real situation….then it will become easier to identify mental illnesses at primary care level”*

*“All the staff in hospitals including the nurses, attendants and other staff and other non clinical staff should get proper awareness about these problems (mental health) and they should be trained to deal with and tackle a patient with a mental illnesses. As an initial step we can start with training the heads of the institutions or the medical officers or nursing officers because these people have a higher level of education. Once they are trained they will train their subordinates”*

*“In primary care set up, develop a training program for medical officers and public health workers in our area to screen and to identify mental disorders”*

*“Other health workers also should get training and awareness about mental health”*

*“We should train and use the public health inspectors to give awareness programme to our staff including midwives and family health assistants, then we encourage them to conduct seminars or workshops at community level and in clinics”*

*“Guidelines…..on how to treat the people in mental disorders and how to handle people are needed…because in Sri Lanka..in primary care institutions….there are no guidelines on how to treat people with mental disorders… there is no policy….I think it is better to have a policy”*

Integration of mental health into primary care was seen as a much needed initiative for the region. However, the PCP felt that this integration should consider existing care burden at this level.

*“We don’t have any data on the existing burden….we need more data on mental illness burden”*

*“We have to take a multi*-*sectoral approach to integrate all services at primary care level”*

*“Integrating mental health care to GP (general practitioner) setup or family health physician set up is good for providing better quality service to people”*

In addition, integration of mental health training and screening to the national non-communicable disease prevention plans were recommended by PCP. Another recommendation was to include mental health training programmes in post-graduate medical education in family medicine, and to use more accessible teaching methods such as online courses.

*“We can get funds. We can get drugs. The thing we don’t have is sufficient man power…. I believe that mental health or screening for mental illnesses should be incorporated into our national non communicable disease programme….to incorporate that I think government should pay attention….currently these non*-*communicable disease screening programmes is limited to heart diseases and other vascular diseases…..the government strategies seem less concerned about mental health”*

*“To make it (integration of mental health into primary care) successful we should have adequate staff at this level…..have to mobilize all the mental health care resources….we should train more psychiatrists and….more should be done at…I think..post graduate medical education level. Have to recruit more psychiatrists…..so that they can be employed at base hospitals at least. Now we have psychiatrists at teaching hospitals….We should have at least one mental health officer at divisional hospitals”*

*“In the GP setup we can arrange some online courses…..similar to diploma in family practice…..so doctors from faraway places…especially GPs can update their knowledge and get an additional qualification….. and improve the quality of their services”*

## Discussion

The COMGAP study was planned as a local demonstrative project exploring the feasibility of training PCP in order to improve the integration of mental health into primary care. This paper presents combined outcomes of the pilot study that included a training programme and a qualitative component. To our knowledge, this is the first instance that the WHO mhGAP IG has been used in Sri Lanka to train primary care practitioners, especially in the post-conflict context to improve primary mental health care. We report a post-training improvement of mental health knowledge/skills among participants, feedback on adaptation requirements of mhGAP IG and views/perceptions of PCP on integrating mental health into primary care in a post-conflict region. In addition, valuable lessons about the challenges involved in implementing mental health training interventions in resource-poor LAMIC settings were also learnt.

Findings provide and insight into the post-conflict population profiles, their mental health needs as well as mental health care provision at primary care levels. Qualitative findings clearly highlight the fact that despite the significant levels of mental health issues among displaced and returnee post-conflict populations in the region, service provision is lacking at primary care level. According to our findings, this is due to many factors; increased patient loads at primary care facilities, lack of mental health-related training for PCP, stigma, lack of awareness among communities as well as health workers, lack of human resources at health facilities and lack of guidelines/policy on mental health at primary care level. As emphasized by PCP, other public health personnel (PHP) at primary care level such as public health midwives (PHM) or support workers do not feature in any current mental health care provision scheme, despite their close link to communities. Some of the current findings echo issues reported by Nagai et al. 2007, and shows that much needs to be done to address underlying health infrastructure problems in Sri Lankan post-conflict regions [9, 10]. In addition, the lack of clear epidemiological evidence about the mental health disorder burden at primary care level has been highlighted.

Over the last decade or so, the Sri Lankan mental health system has undergone substantial reforms with more diverse mental health services available at district level [11]. Since the 2004 tsunami, importance of mental health services at primary care level has been increasingly recognized [11]. A national mental health policy has been established, training programmes have been conducted and mental health services have been expanded [11]. However, these activities have not been coordinated to create an overarching programme for integrating mental health into primary care and PCP were not part of this reform, especially for post-conflict populations. More recently, Jenkins et al. [2012] reported a ‘train the trainers’ programme that aimed to develop a sustainable partnership between psychiatrists, public health system and private sector to promote the integration of mental health into primary care. However, this training programme has its limitations as it did not sufficiently evaluate long-term outcomes and was primarily aimed at doctors (psychiatrists and other cadres). It also did not focus specifically on post-conflict mental health needs. This programme was conducted before the introduction of mhGAP by the WHO [14]. Qualitative findings from that programme are similar in many aspects to current study results, where increased patient loads and lack of resources are cited as barriers, participants recommend training all health cadres in mental health, indicate the need to have role-play/training materials in local languages and highlight an overall need for training programmes [14]. Our attempts through this study were to address the gaps in available mental health services and explore the feasibility of potential solutions such as engaging more PCP, especially in the post-conflict Northern Province.

At the global level, much attention has been paid to the topic of integrating mental health into primary care, including for post-conflict and other humanitarian settings, despite numerous difficulties faced during the practical implementation process [3, 15–18]. Our qualitative findings are broadly reflective of other work conducted in different global regions on qualitative evaluation of mental health integration to primary care [19]. The mhGAP programme is currently considered as the frontline tool for developing mental health at primary care level and is widely used, despite a number of limitations [2]. Various studies have been conducted in Asia, Africa and other parts of the world, with mhGAP IG utilized as central part of training interventions for primary or community health care workers [2, 16, 20, 21]. However, only limited evidence is available on training interventions that focus on integrating mental health into primary care exclusively for post-conflict (which can have displaced/returnee populations) settings [15], with mhGAP IG as the main training guide. The current study, to our knowledge, is the first to report findings on feasibility of using mhGAP in a post-conflict, resource-poor setting. Our findings, although on a preliminary level, provide further understanding about the implementation aspects of mhGAP. Our findings highlight the lack of culturally/linguistically relevant mhGAP training material, and point out the practical limitations of engaging PCP from congested and under-resourced primary care facilities to match the existing requirements of mhGAP delivery [2]. More research is required to explore potential scaling-up of mhGAP-based training interventions in post-conflict settings to integrate mental health with primary care.

### Study limitations and local implementation challenges

Limitations include low numbers of participants, both for the training and the qualitative component, limited evaluation (only through pre/post testing), lack of post-training monitoring mechanisms, selective use of mhGAP modules and using only a single trainer. Some of these limitations are due to factors beyond our control, and are related to implementation challenges. As noted earlier, this study was planned as a pilot, feasibility study on a limited budget, and therefore had inherent limitations within the design. Further scaling-up of the intervention will enable a more robust methodological execution, enabling long-term post-intervention monitoring and evaluation.

As mentioned in the methodology section, we faced a number of challenges during the implementation, which had a critical impact on study conduct and final outcomes. Some of these challenges are unique to Sri Lanka, as they are intricately connected to the public health system and its administrative aspects. Other challenges may be more common to resource-poor, LAMIC settings, compounded by post-conflict contextual factors. We believe that these challenges and our strategies to address them warrant a longer discussion, especially to highlight country-specific and LAMIC barriers towards the integration of mental health into primary care through training interventions.

#### Support from administrative bodies and stakeholders

Support from administrative bodies and key stakeholders such as the Ministry of Health, Provincial and regional health authorities and hospital administrators were crucial for the success of the study. However, we had mixed responses from key decision makers at various levels. Some were highly supportive, while engaging others took considerable effort and caused delays with the study schedule.

#### Interest and support from PCP

A lack of interest was observed during the initial recruitment period from PCP when they were approached to take part in the study. We even had to engage a prominent doctors’ trade union to overcome initial resistance. A similar obstacle was presented when organizing the delivery of the training intervention. Approached individually, most PCP indicated a clear reluctance, citing workload, lack of time, difficulty with travel or issues with compensation (as most PCP do private practice beyond their government duties, they indicated a loss of income if they took part in the training). To mitigate this lack of interest, central and provincial health authorities had to be involved. However, despite the mitigating action by provincial authorities in nominating and selecting potential participants, only a limited number of PCP participated in the full 3 day training.

#### Participant retention

Another significant challenge, which ultimately led to changes in how the study was conducted and created questions on feasibility, was the retention of recruited PCP. A number of PCP were recruited at the start of the planned pilot RCT in early 2014. However, within few weeks, all those recruited had dropped out of the study. A key reason for this was the high turnover of PCP in the region. Most PCP serving in these regions are early post-internship doctors (in Sri Lanka, every medical graduate has to serve an internship programme at a tertiary-level hospital upon completion of medical school, and are subsequently placed as post-internship medical officers for another 2 years at rural primary care units). These medical officers receive regular, periodic transfers leading to a high staff turnover at the primary care facilities in the region. Majority of PCP recruited belonged to this category and had received transfers shortly after enrolling in the study. This high staff turnover is a major challenge in conducting RCT or intervention programmes involving training components and has a substantial impact on any meaningful evaluation. Most primary care facilities in Sri Lanka (especially post-conflict and rural areas) are staffed with similar cadres, presenting significant challenges for potential scaling-up of training interventions.

Other challenges in retaining PCP in the study included increased workloads and lack of human resources. Most PCP in the primary care units in the region have very high patient loads, sometimes as high as 200 patients per day (also cited by PCP in the qualitative component). This is due to the lack of adequate numbers of medical officers/primary care units in the region. This increased workload prevented the PCP from dedicating sufficient time for study-related activity. We were informed that lack of manpower also prevented some PCP from participating in the training programme, as there were no replacements to oversee the clinical workload in their absence. For example, according to one PCP, few villages falling under his responsibility would go without the services of a single doctor if he was to take part in our training. Even though he was willing to attend the training, he could not leave his clinic unattended for the 3 day period required for course, without seriously jeopardizing his professional commitments and the trust of the population.

#### Geo-political issues

Northern Sri Lanka is a region that experienced a 30 year civil conflict. After the cessation of conflict in 2009, this post-conflict region has been undergoing a massive regeneration and rehabilitation effort. Civil society changes have been also taking place. The first election in the region after three decades of conflict took place in early 2014, for the Northern provincial council. This election and preparations for it was coincided with the start of COMGAP study preparations and recruitment. Due to restrictions of movement and security issues posed to the study team, COMGAP commencement was postponed until the election was over and the region regained normality. In addition, there were number of seasonal weather events that affected the region including large scale floods, which caused delays during various phases of the study.

#### Resources

During the training programme, we experienced a number of issues with the available course resources. The mhGAP has several videos and other assistive material available and these were used during the training. However, these material had been developed for other settings and as shown in results from the qualitative interviews with PCP, participants indicated that material linked to the cultural and background context (post-conflict region with high levels of trauma, even among course participants), would have been beneficial.

## Conclusion

COMGAP pilot study was planned to explore the feasibility and uptake of integrating mental health into primary care by training PCP serving a post-conflict population. Important lessons were learnt about the feasibility, especially about engaging PCP in such an initiative, involvement of key stakeholders, retention of study participants and the delivery of training in post-conflict settings. Attitudes and perceptions of PCP on integrating mental health into primary care were explored. It should be noted that the study cannot be interpreted as a failed trial attempt as we had to face ground realities with the implementation aspects. The study process has provided us with valuable lessons on planning research, especially RCTs in primary care settings in Sri Lanka. While the top down approach of engaging the policy planners was successful, main issue was engaging busy primary care practitioners on the ground. This prompts a need for strategic re-think around recruitment and motivation of PCP for future interventions.

Our study has provided important insights into the requirements for potential scaling-up. It has also provided information on the WHO mhGAP intervention guide and teaching resources, and will lead into the development of locally relevant material, which can then be incorporated into the existing pool of global resources. Broader lessons learnt during the study would be helpful in planning similar interventions among other global conflict-affected populations and humanitarian contexts.

With the relative political, social and economic stability in the northern region after three decades of bitter conflict, training new cadres of healthcare workers and increasing the capacity of existing health work force is of critical importance to address future mental health needs of Sri Lanka. Currently, plans are underway to scale-up COMGAP study in Northern Sri Lanka and to include primary care practitioners as well as public health workers. The scaling-up will utilize lessons-learnt during the pilot phase. Increasing knowledge, skills and awareness about mental illnesses between front-line PCP and other health workers may improve treatment practices and decrease regional burden of mental disorders. In addition to local benefits, current findings add to the global mental health evidence base.
